# Microbiome and Cancers of the Esophagus: A Review

**DOI:** 10.3390/microorganisms9081764

**Published:** 2021-08-18

**Authors:** Yukiko Yano, Arash Etemadi, Christian C. Abnet

**Affiliations:** Division of Cancer Epidemiology and Genetics, National Cancer Institute, National Institutes of Health, Bethesda, MD 20892, USA; arash.etemadi@nih.gov (A.E.); abnetc@mail.nih.gov (C.C.A.)

**Keywords:** oral microbiome, esophageal microbiome, esophageal cancer, esophageal squamous cell carcinoma, esophageal adenocarcinoma, oral health

## Abstract

Esophageal cancer (EC) is an aggressive malignant disease ranking amongst the leading causes of cancer deaths in the world. The two main histologic subtypes, esophageal squamous cell carcinoma (ESCC) and esophageal adenocarcinoma (EAC), have distinct geographic and temporal patterns and risk factor profiles. Despite decades of research, the factors underlying these geo-temporal patterns are still not fully understood. The human microbiome has recently been implicated in various health conditions and disease, and it is possible that the microbiome may play an important role in the etiology of EC. Although studies of the microbiome and EC are still in their early stages, we review our current understanding of the potential links between ESCC, EAC, and bacterial communities in the oral cavity and esophagus. We also provide a summary of the epidemiology of EC and highlight some key challenges and future directions.

## 1. Introduction

Esophageal cancer (EC) is the seventh most common incident cancer and the sixth leading cause of cancer mortality in the world [1]. Esophageal squamous cell carcinoma (ESCC) is the predominant histologic type of EC (84% of all cases), followed by esophageal adenocarcinoma (EAC, 15% of all cases) [2]. Whereas EAC tends to occur in the distal esophagus, ESCC can be found throughout the esophagus [3]. The two histologic types also exhibit notable differences in geographic patterns, time trends, and risk factor profiles [4,5]. Since symptoms appear late and patients are often diagnosed at an advanced stage, both ESCC and EAC have a poor prognosis, with a median survival of 10 months and a 5-year survival rate of 22% in the United States (US) [6], but this value is lower in the low- and middle-income countries where this cancer is most common. Primary prevention and early detection are key to reduce the global burden of EC, but these efforts are hampered by the knowledge gap that remains about the etiologies of ESCC and EAC. 

The advent of culture-independent, high-throughput DNA sequencing along with advanced computational tools have greatly enhanced our understanding of the complexity of the human microbiome and how the microbiome may be involved in the development of various diseases, including cancer [7,8]. The human microbiome refers to the collection of microorganisms, including bacteria, archaea, fungi, and viruses, inhabiting the human body. These microbial communities, particularly bacteria, have co-evolved with the host to play beneficial roles in nutrition, immune function, and metabolism [9]. In contrast, there is also increasing evidence suggesting that the microbiome may contribute to carcinogenesis, specifically through its key roles in metabolism and inflammation [10]. Although the literature is still relatively limited for EC, recent studies suggest that the microbiome may also be involved in the etiology of EC. 

Here, we review the current knowledge in the epidemiology of ESCC and EAC, and we highlight potential avenues for future etiologic investigation, particularly pertaining to the human oral and esophageal microbiome. This review focuses on studies of the link between the bacterial communities and EC in humans. Other microorganisms, including archaea, fungi, protozoa, and viruses are not discussed. The purpose of this review is to provide readers a comprehensive overview of the current evidence exploring the potential connection between the oral and esophageal microbiome and esophageal cancer. 

## 2. Methods

We performed an electronic search using PubMed and Web of Science (until March 2021) databases with the following keywords: esophageal cancer, esophageal squamous cell carcinoma, esophageal adenocarcinoma, oral microbiome, esophageal microbiome, bacteria, pathogen, oral health, tooth loss, tooth decay, periodontal disease, periodontitis, gum disease, oral hygiene, brushing, fluorosis. We also searched references of selected articles. Only articles written in the English language were included. We did not use exclusion criteria due to the limited number of existing articles related to this topic.

## 3. Epidemiology of EC

### 3.1. Descriptive Epidemiology of EC

ESCC shows striking geographic variation in incidence globally, with more than 10-fold differences between countries [2,4]. The highest incidence rates are observed in two geographic belts—one from Eastern to Central Asia (the “Asian Esophageal Cancer Belt”), and another along the Rift Valley in East Africa and into South Africa (the “African Esophageal Cancer Corridor) [4]. In 2018, more than 80% of ESCC cases were concentrated in Asia (most of which occurred in China), and the highest age-standardized incidence rates were observed in countries such as Mongolia, Malawi, and Kenya (>16 per 100,000 person-years) [2]. While the burden of ESCC remains high in these regions, ESCC incidence has been declining or stabilizing in other parts of the world, such as the US [11] and Europe [12], during the last several decades. 

In many of the high-income countries in Europe, North America, and Oceania, where ESCC rates have been declining, there has been a concurrent rapid increase in EAC incidence over the past 30 years [5]. The highest incidence rates were observed in the Netherlands and the United Kingdom (UK) in 2018 (age-standardized incidence rate of 4.4 per 100,000 person-years) [2]. ESCC is currently the most common subtype of EC in the world, but the incidence rate of EAC has surpassed that of ESCC in a number of countries (e.g., the UK, The Netherlands, the US, Sweden, Canada, Australia) [2,13,14], and this trend of increasing EAC and decreasing ESCC rates is predicted to continue across an expanding number of high-income countries [15]. 

These geographic and temporal variations in the incidence of EC suggest that modifiable risk factors may be involved in the etiology of ESCC and EAC, and changes in the prevalence of these risk factors may contribute to the variations seen in EC incidence [16,17,18].

### 3.2. Risk Factors for EC

The etiology of ESCC is multifactorial, and the primary risk factors appear to be population-dependent [4]. Tobacco smoking and alcohol consumption are established causal factors for ESCC [19] that account for the majority of cases in high-income countries [16,20,21]. However, tobacco smoking and alcohol consumption do not explain the burden of ESCC in high-risk areas, where these practices are relatively uncommon, or they contribute modestly to ESCC risk [4,22,23]. Extensive epidemiologic research conducted in ESCC hotspots such as Linxian, China [24,25], and Golestan, Iran [26,27], and more recent studies in Africa [28,29] have provided important insight into additional risk factors, which are often more present in lower-income populations that are disproportionately impacted by this fatal disease [30]. These additional risk factors include nutritional deficiencies (e.g., selenium) [31], possibly mycotoxin contamination of food (e.g., pickled vegetables [32]), consumption of hot beverages and food [33] (e.g., mate [34] and tea [35,36,37]), exposures to polycyclic aromatic hydrocarbons (e.g., from biomass burning used for cooking and heating [38,39]), opium use [40,41], betel quid chewing [42], and drinking un-piped water [24,43,44]. Family history of ESCC has been shown to increase risk, but the role of genetic factors in ESCC etiology is not well understood [4,30,45]. An inverse association between socioeconomic status and ESCC has been consistently reported in both high-income countries and in high-incidence/lower-income regions [43,46,47], but the underlying factors driving this relationship are unclear [4,22,48]. Poor oral health conditions resulting from shifts in the oral microbiome are another potentially important risk factor that has been consistently associated with ESCC risk, and this will be discussed in detail in the following section.

Risk factors for EAC differ markedly from those of ESCC. The two strongest risk factors for EAC are gastroesophageal reflux disease (GERD) and obesity [5], and the combination of these two risk factors together with smoking may account for over 70% of EAC cases in Western countries [16,49]. GERD is a common gastrointestinal disorder that involves reflux of stomach contents into the esophagus or mouth, causing symptoms such as heartburn and regurgitation [50]. Individuals with chronic GERD are at an increased risk of developing the precursor lesion for EAC, Barrett’s esophagus (BE), in which the squamous epithelium of the esophageal mucosa undergoes metaplasia to columnar intestinal epithelium [51]. The presence of BE is associated with a 10- to 40-fold higher risk of EAC [52], but only a small proportion of patients with BE progress to EAC (0.1 to 0.3% per year) [53]. Obesity has been consistently associated with an increased risk of EAC, with risk increasing linearly with higher BMI [54,55,56]. Tobacco smoking is a moderately strong risk factor for EAC (2-fold increased risk in ever vs. never-smokers) [57], whereas alcohol is not associated with EAC risk [58,59]. Although genetic factors contribute to the etiology of EAC [60], shifts in the prevalence of key risk factors are likely to have driven the sudden increase in incidence across Western populations [5,61]. In the US, EAC incidence began to increase dramatically in the 1970s [62], and parallel increases in obesity and GERD may have contributed, but other factors are likely to be involved [52,61]. One hypothesis is that the rise in EAC may be related to the decline in infection rates of *Helicobacter pylori* [61]. *H. pylori* is a Gram-negative bacterium residing in the stomachs of about half the world’s population [63], causes most cases of gastric cancer, and is currently the only bacterium judged to be a group 1 carcinogen by the International Agency for Research on Cancer (IARC) [64]. However, *H. pylori* has consistently been shown to be inversely associated with EAC, with a nearly 50% reduction in risk [65,66]. The widespread use of antibiotics in the 1940s, along with the decline in *H. pylori* infection rates in the 1950s, may have led to large-scale changes in the human microbiome leading to the rapid increase in EAC [52].

### 3.3. Poor Oral Health as a Risk Factor for EC

Poor oral health, indicated by periodontal disease and tooth loss/decay, is a potentially important and preventable risk factor that involves shifts in the oral microbiome that may contribute to carcinogenesis in the esophagus. In fact, poor oral hygiene has been identified as one of the agents recommended for evaluation, with high priority for an upcoming IARC Monographs on the identification of carcinogenic hazards to humans [67]. Oral health—assessed by tooth loss; the sum of decayed, missing, or filled teeth (DMFT score); periodontal health; and oral hygiene practices (e.g., tooth brushing)—has been examined as a risk factor for ESCC in numerous epidemiologic studies [68,69,70,71,72,73,74,75,76,77,78]. A positive association between tooth loss and ESCC risk has been found repeatedly in both case-control and large-scale prospective cohort studies conducted in Linxian [68,69,70] and Golestan [73,74]. Similar findings have been found in other high-risk areas such as Kashmir, India [72], Taixing, China [71], and Kenya [75,76], in addition to Japan [77] and parts of Latin America and Europe [78]. Regular tooth brushing has been shown to have a protective effect against ESCC in various studies [71,72,73,74]. Recent meta-analyses suggest an odds ratio (OR) of 1.3 to 1.5 comparing the highest versus lowest number of teeth lost [79,80,81] and an OR of around 0.60 when comparing high- versus low-frequency of tooth brushing [79,82] for overall EC risk (estimates were slightly weaker for tooth loss [79,80] and slightly stronger for tooth brushing [79] when the analysis was restricted to ESCC). Periodontal disease has also been associated with an increase in overall EC risk (no histological distinctions) [83,84]. Furthermore, two recent case-control studies from East Africa have shown that dental fluorosis, an irreversible hypo-mineralization of the tooth enamel characterized by brown stains and pits in severe cases, may be an important understudied risk factor contributing to ESCC risk in this high-risk region. Moderate/severe dental fluorosis was strongly associated with ESCC risk (compared with no dental fluorosis) with an OR of 14.7 (95% confidence interval (CI): 7.6–28.6) [76] and 13.5 (95% CI: 5.7–31.9) [85] in case-control studies from Kenya and Tanzania, respectively. The large effect sizes and consistent observations across these two independent studies warrant further investigations to assess how dental fluorosis may impact ESCC risk.

In contrast, there have also been some studies reporting null associations between poor oral health and EC risk. Null associations have been found in prospective studies conducted in Finland [86,87], Korea [88], the UK [89], Taiwan [90,91], and among male health professionals in the US [92]. With the exception of one study [86], these studies did not distinguish between ESCC and EAC, and this may have inhibited the ability to detect risk associations since poor oral health may have distinct effects on the two histologic subtypes. Although the reasons for the inconsistent findings are unclear, it is also possible that the relative importance of poor oral health as a risk factor for EC, particularly ESCC, may differ between high-incidence regions (e.g., Linxian and Golestan) and areas with lower incidence, which are oftentimes in high-income countries that tend to have better overall oral health (e.g., the US and the UK). 

There has only been one study to date that specifically evaluated the relationship between poor oral health and EAC [93]. In a prospective investigation of men and women in the US, this study showed that periodontal disease history was associated with a hazard ratio (HR) of 1.43 (95% CI: 1.05–1.96) and that having lost two or more teeth was modestly associated with increased EAC risk (HR 1.42, 95% CI: 1.00–2.03) compared with having no lost teeth. Having periodontal disease and losing at least one tooth was associated with 1.59-times higher EAC risk (95% CI: 1.04–2.44) compared with having no periodontal disease and no tooth loss. Clearly, additional studies are needed to better understand the relationship between poor oral health and EAC. 

The existing epidemiologic evidence indicates that there may be a link between oral health and EC, but the underlying mechanism for the association is not understood. It is well established that oral bacteria are directly involved in the development of periodontal disease (which often results in tooth loss) and dental caries, the two most prevalent oral disorders worldwide [94]. Therefore, it is hypothesized that the human oral microbiome may also be involved in the etiology of EC [95], which will be discussed in later sections of this review. 

## 4. Oral Microbiome

### 4.1. Oral Microbiome in Health

The oral microbiome is one of the most complex body habitats, with at least 700 bacterial species identified in the oral cavity [96,97]. Oral bacterial communities are particularly diverse in terms of community membership and have high alpha (within-sample) diversity (i.e., measured by species richness (number of taxa) and evenness (equal abundance)) compared with other body habitats [98,99]. The oral microbiome has also been found to have one of the largest core taxa (microbes commonly shared among individuals) and low beta (between-sample) diversity compared with other body habitats—i.e., although each person has an ecologically rich oral microbiome, similar microbes are present in individuals from the same population [98,99,100,101,102]. The six major phyla—Firmicutes, Bacteroidetes, Proteobacteria, Actinobacteria, Spirochaetes, and Fusobacteria—account for 96% of the taxa detected in the oral bacterial community [96]. A recent study of more than 1000 healthy adults showed that the oral microbiome was relatively stable between individuals at the genus level (although there was substantial variation at higher taxonomic resolution), with 11 core genera present in more than 99% of all individuals, which together accounted for approximately 78% of the total relative abundance in the bacterial community [103]. The core genera consisted of *Streptococcus*, *Veillonella*, *Gemella*, and *Granulicatella* of the phylum Firmicutes; *Neisseria* and *Haemophilus* of Proteobacteria; *Prevotella* of Bacteroidetes; *Actinomyces* of Actinobacteria; and *Fusobacterium* and *Leptotrichia* of Fusobacteria, with some of these genera present at relatively low abundance (mean relative abundance < 2%). Although there is no clear definition of what a normal or healthy oral microbiome looks like [104], it is possible that this core oral microbiome seen in health may be altered in the presence of disease [105]. 

Oral microbes play important roles in both oral health and overall systemic health. The resident oral microbiome is capable of inhibiting colonization by pathogens, as well as being involved in the maturation of the host innate and adaptive immune systems, and some oral bacteria have immunomodulatory roles in maintaining appropriate host immune responses and in maintaining host–microbe homeostasis [106,107,108]. It is when this symbiotic relationship between the host and the oral microbiome break down that oral diseases are thought to occur [106], and this may also have implications for systemic diseases, including cancer [109,110]. 

### 4.2. Factors That Impact the Oral Microbiome

The dynamic and diverse oral microbiome is shaped by various factors. Early microbial colonization of the oral cavity occurs progressively from birth, with microbes acquired from the birth canal, breast milk, and the mother’s mouth [108,111]. Pioneer microorganisms colonizing a newborn’s mouth are usually Gram-positive cocci, including *Streptococcus* and *Staphylococcus*, and these initial colonizers then condition the oral environment for subsequent colonization by other species [111,112]. The oral microbiome becomes more complex and diverse during the first few years of life, and important alterations occur during the eruption of primary and permanent teeth, which present new niches (i.e., hard surfaces of teeth and gingival crevices) for microbial colonization [111]. Alterations of the oral microbiome occur at other life stages, including during puberty, when hormone levels change, and in old age, when all teeth are lost and the bacterial community resembles that of children before tooth eruption [111]. Use of prosthodontic (e.g., dentures) [113] and orthodontic appliances have also been shown to have profound impacts on the oral microbiome [114,115]. In addition to age, other factors are suspected to impact the oral microbiome. Host genetics contribute in shaping the oral microbiome, but twin studies suggest environmental and lifestyle factors are the main drivers in shaping oral microbial communities [116,117,118]. Previous studies have examined the impact of various sociodemographic and lifestyle variables, including diet [119], geography [120], cohabitation [121], and race/ethnicity [120,122], but current evidence suggests these factors may only account for a small proportion of the inter-individual variation seen in the oral microbiome [103]. Perhaps one of the most important factors that modifies the oral microbiome is oral health [119]. Epidemiological evidence suggests poor oral health is linked to EC risk, and this relationship may involve alterations in the oral microbiome related to poor oral health conditions.

### 4.3. Oral Microbiome and Oral Health

The link between poor oral health and esophageal cancer is likely to involve alterations in the diverse community of microbes present in the oral cavity. There are several distinct microbial niches within the oral cavity, including saliva, soft tissue surfaces of the oral mucosa and tongue, and hard tissue surfaces of teeth [123]. Different habitats sampled within the oral cavity were found to form three different bacterial community groups: (1) buccal mucosa (cheeks), keratinized gingiva (gums), and hard palate; (2) saliva, tongue, tonsils, and throat; and finally (3) supra- and subgingival (above and below the gum) plaque, which were found to be particularly distinct from the other oral sites [124]. In contrast to the continuously shedding superficial epithelial layers of the oral mucosa, the non-shedding hard surfaces of teeth (as well as prosthodontic/orthodontic appliances) provide a stable location for the long-term formation of dental plaque, a complex microbial biofilm [125]. The salivary microbiome includes microbes detached from various niches in the oral cavity and may be representative of the overall oral microbiome [126]. It is now recognized that dental caries and periodontal disease, the two most common oral diseases, result from shifts in the consortia of microbes present in the supra- and subgingival biofilm communities, respectively [94].

The development of dental caries and periodontal disease both involve interactions between the host and complex polymicrobial communities in oral biofilms [94]. Caries result from demineralization of enamel and breakdown of the tooth surface by acid produced through fermentation of dietary carbohydrates (sugar) by oral bacteria [94,97]. In the presence of frequent exposure to sugar, acidogenic and aciduric bacteria accumulate in the biofilm matrix, leading to increased acid production. As long as the biofilm is not removed (by means of oral hygiene practices) and frequent exposure to sugar persists, acidification of the localized region will continue, eventually resulting in enamel demineralization [94]. For decades, it was thought that *Streptococcus mutans* was the primary etiological agent in dental caries [125]. However, more recent molecular studies have revealed a much larger community of bacteria associated with caries, including non-streptococcal bacteria that were once considered non-cariogenic, such as *Bifidobacterium* spp., *Actinomyces* spp., and *Enterococcus faecalis* [106,127].

Periodontal disease involves the interplay between oral microbiome and the host immune response. Gingivitis entails mild and reversible inflammation of the gums, whereas periodontitis involves tissue destruction and loss of tooth support [94,128]. In periodontitis, a profound shift in the composition of subgingival communities occurs, with an enrichment of Gram-negative anaerobic bacteria, and these changes in the subgingival microbiome associated with periodontitis development are markedly different from those in gingivitis [128]. Among those enriched are the triad of *Porphyromonas gingivalis*, *Treponema denticola*, and *Tannerella forsythia* (known as the “red complex”), which were classically considered as the predominant pathogens in periodontitis [94,125,129]. Culture-independent analyses have expanded this list of periodontitis-associated taxa enriched in disease to include *Porphyromonas endodontalis*, several *Treponema* spp., *Prevotella intermedia*, *Filifactor alocis*, *Fretibacterium* spp., and *Selenomonas* spp., among others [125,128,129]. In addition, *Actinomyces* spp., *Rothia* spp., and *Streptococcus sanguinis* are the main health-associated taxa that are often depleted in periodontitis, and *Campylobacter gracilis* and *Fusobacterium nucleatum* ss. *vincentii* are the two core species consistently detected in health and in periodontitis [129]. Periodontitis is associated with an increase in richness of community membership compared with health, probably due to the availability of additional nutrients derived from host tissue damage and also due to the increased physical space in the gingival crevice [94]. Inflammation may be a key driver of the overgrowth of periodontitis microorganisms through tissue destruction providing a rich source of nutrients, and these bacteria can further promote inflammation to maintain their selective dominance [94,130]. Many of the periodontitis-related pathogens are also present in health, rather than being exogenous pathogens absent in health—it appears that some species become more dominant in a disease state, although they are also present at low levels in health [94,128]. For example, *P. gingivalis* is a minor member of the oral microbial community, but it is consistently associated with the initiation and progression of periodontal disease. However, *P. gingivalis* alone does not appear to induce periodontal disease, and so it is thought that *P. gingivalis* may act as a keystone pathogen that changes the environment to alter the growth of other microorganisms in the community [125]. It is hypothesized that this may lead to the emergence of a polymicrobial community in which members act synergistically and interact with the host immune response to cause periodontal disease [94,125].

The main mechanism by which the oral microbiome is thought to contribute to carcinogenesis at distant sites is through systemic inflammation, possibly triggered by periodontal disease [95,131]. Periodontitis impacts both local and systemic immune responses [132], and inflammation is a critical hallmark of carcinogenesis [10,133]. Periodontal disease involves ulceration of the gingival sulcular epithelium, which allows oral microorganisms to translocate into the bloodstream, leading to systemic dissemination of oral pathogens that may cause infections and inflammation in distant sites of the body [95,131,134]. In addition, oral bacteria can contribute to carcinogenesis through local activation of carcinogens such as acetaldehyde and nitrosamines [95,132]. It is possible that oral bacteria may contribute to carcinogenesis in the esophagus through these mechanisms, and there are some studies that have found associations between the oral microbiome and EC risk.

## 5. Oral Microbiome and EC

### 5.1. Oral Microbiome and ESCC

A few cross-sectional case-control studies have characterized the differences in the microbiome of ESCC patients and healthy controls (Table 1). Chen et al. compared the salivary bacterial microbiome of 87 ESCC cases, 63 subjects with esophageal squamous dysplasia (ESD, the precursor lesion for ESCC), and 85 healthy controls from Taixing, China [135]. In this study, ESCC cases had an overall decreased alpha diversity (measured by species richness and Shannon and Chao1 indices) compared with the dysplasia and control subjects, and principal coordinates analysis (PCoA) of beta diversity matrices (weighted and unweighted UniFrac) showed that ESCC cases and controls separated from each other, with ESD subjects in between the two. When looking at taxa-specific differences, relative abundances of the genera *Prevotella* (*P* adjusted for multiple comparisons (adj *p* ≤ 0.04)), *Streptococcus* (adj *p* < 0.01), and *Porphyromonas* (adj *p* ≤ 0.02) were significantly higher, and most other genera were lower in ESCC cases compared with the control and dysplasia subjects. The proportion of *Prevotella* and *Streptococcus* accounted for nearly 65% of the overall community in ESCC subjects, whereas these two genera comprised about half of the bacterial community in non-ESCC subjects. In contrast, two other studies by Wang et al. [136] and Zhao et al. [137] found no significant differences in alpha diversity between ESCC cases and healthy controls in the salivary microbiome of Chinese individuals. When comparing 20 ESCC cases with 21 controls, Wang et al. found that the ESCC group had higher relative abundances of the genera *Actinomyces* and *Atopobium* and lower relative abundances of *Fusobacterium* and *Porphyromonas* compared with the control group, although none of these taxa were statistically significant after multiple testing correction [136]. While there was no difference in alpha diversity, analyses of beta diversity (weighted UniFrac) showed noticeable differences in the community composition of 39 EC cases (33 ESCC, 6 EAC) and 51 healthy controls in the study by Zhao et al. [137]. This study also found a number of differentially abundant taxa, including *Prevotella* (adj *p* = 0.0704) and *Neisseria* (adj *p* = 0.0013), which were higher and lower, respectively, in EC cases compared with controls, and this was also observed by Chen et al. [135]. Although there have only been a few studies, all with small sample sizes, these studies show that the composition of the oral microbiome may differ in those with and without ESCC. 

Two prospective case-control studies have provided insight into how the oral microbiome relates to the etiology of ESCC (Table 1). Liu et al. conducted a nested case-control study in China using prediagnostic oral swab specimens collected from 84 cases with esophageal lesions of severe dysplasia and above (including severe squamous dysplasia, carcinoma in situ, and ESCC) and 168 matched healthy controls [138]. In this study, cases had slightly higher alpha diversity (as measured by the Shannon index, *p* = 0.044), but there was no difference in beta diversity between the two groups. This study also identified 11 species that may be predictive of the risk for malignant esophageal lesions, which included *Actinomyces* spp., *Dialister invisus*, *Fusobacterium* spp. (including *F. nucleatum*), *Leptotrichia hofstadii, Prevotella* spp., *Rothia dentocariosa*, and *Lachnoanaerobaculum umeaense*—all of which were present at higher relative abundances in cases. While all abovementioned studies were from Chinese populations, Peters et al. examined the oral microbiome in a US population using prediagnostic mouthwash samples collected from 25 ESCC cases and 50 matched controls [139]. ESCC cases did not differ significantly from controls in alpha or beta diversity in this study. Interestingly, the periodontal pathogen *P. gingivalis* was modestly associated with ESCC risk (OR = 1.30, 95% CI: 0.96–1.77, *p* = 0.09), and several other species showed nominal association with ESCC (none were significant after multiple testing correction): Higher abundance of *Prevotella nanceiensis*, *Bergeyella oral taxon 322*, *Neisseria weaveri*, and *Treponema vincentii* was associated with increased risk, while higher abundance of *Prevotella oral taxon 306* and *Aggregatibacter paraphrophilus* was associated with decreased risk. 

Studies of the oral microbiome and ESCC are at an early stage, and additional studies are clearly needed. While the findings from existing studies indicate that alterations in the composition of oral bacterial communities may be linked to ESCC—namely, changes in the relative abundance of specific taxa such as *Prevotella* species—these studies are limited by their small sample sizes and mostly cross-sectional design. Cross-sectional studies are informative in describing the differences in the microbiome of individuals with and without EC and for assessing whether these differences have clinical implications on prognosis or survival outcomes. However, these studies cannot assess temporality and are limited in their ability to make inferences about etiology. Reverse causation, where the presence of disease alters the microbiome rather than changes in the microbiome being a causative agent of disease, cannot be ruled out in cross-sectional designs in which microbial exposures and disease outcome are assessed at the same time point. Prospective designs are necessary to evaluate how the microbiome may be related to the causes of EC, and sample collection must precede disease onset to reduce the chances of reverse causation. In addition, low statistical power, due to small sample sizes and/or small effect sizes, may lead to spurious findings and results that may not be replicated in future studies. Furthermore, corrections for multiple comparisons (e.g., false discovery rate and family-wise error) are crucial in microbiome studies to reduce false positive findings. In summary, larger prospective studies from diverse populations and replication of findings in multiple independent studies are needed in the future to understand the involvement of the oral microbiome in the etiology of ESCC.

### 5.2. Oral Microbiome and EAC

The relationship between the oral microbiome and EAC has been largely unexplored (Table 1). Snider et al. compared the salivary microbiome in a case-control study of 32 patients with BE (including 16 without dysplasia, 6 with low-grade dysplasia, 5 with high-grade dysplasia, and 5 with EAC) and 17 patients without BE who were scheduled to undergo upper endoscopy for clinical indications [140]. There was no difference in alpha diversity between BE patients and controls, but there was significant separation between the two groups based on the weighted UniFrac beta diversity analysis. At the phylum level, relative abundance of Firmicutes was significantly higher (*p* = 0.005) and Proteobacteria was lower (*p* = 0.02) in BE patients compared with controls. Notable differences in relative abundance were found for *Streptococcus*, *Veillonella*, and *Enterobacteriaceae*, which were higher in BE patients. On the other hand, controls had higher relative abundances of numerous taxa, including the genera *Neisseria*, *Lautropia*, and *Corynebacterium*. Furthermore, when comparing patients with non-dysplastic BE with more advanced neoplasia, BE patients with high-grade dysplasia/EAC had increased relative abundance of the family *Enterobacteriaceae*, which contains a number of Gram-negative bacteria linked to infection and inflammation (e.g., *Escherichia coli*) [140].

The aforementioned prospective case-control study by Peters et al. [139] also compared the oral microbiome of 81 EAC cases and 160 matched controls. Similar to their findings for ESCC, there was no difference in alpha or beta diversity between EAC cases and controls. However, taxa-specific analyses indicated that the periodontal pathogen *Tannerella forsythia* was associated with increased EAC risk (OR = 1.21, 95% CI: 1.01–1.46, *p* = 0.04), while depletion of the genus *Neisseria* and the species *Streptococcus pneumoniae* was associated with decreased EAC risk. Additional studies with larger sample sizes are needed to further explore the potential link between the oral microbiome and EAC.

## 6. Esophageal Microbiome

### 6.1. Esophageal Microbiome in Health

Although the esophagus was thought to be sterile in the past, it is now clear it has its own complex microbiome. The esophageal microbiome is influenced by microorganisms swallowed from the oral cavity and refluxed gastric microbes [141]. Major phyla consistently detected in the esophagus, including Firmicutes, Bacteroidetes, Actinobacteria, Proteobacteria, Fusobacteria, and TM7 [52,142,143], closely resemble those found in the oral microbiome. However, the microbiome of the esophagus appears to be distinct from the oral cavity, with notable differences in the microbial compositions (i.e., relative abundances of taxa) of the two sites [144]. In the “normal” esophagus, *Streptococcus* is the predominant genus, followed by *Prevotella* and *Veillonella* [142,144,145,146]. Furthermore, the microbiome of the upper, middle, and lower segments of the normal esophagus appear to be very similar, with no differences in community membership (alpha diversity) or composition (beta diversity) [143,144]—although this may change in the presence of disease (e.g., BE) [147]. Nonetheless, as with the microbiome of other body sites, the “normal” esophageal microbiome is difficult to define due to the large microbiome variance among apparently healthy individuals [104]. 

Most existing esophageal microbiome studies have used biopsies or brushing samples obtained during upper endoscopy for sampling the esophagus. However, the invasive and cost-prohibitive nature of endoscopy has limited many of these previous studies to small sample sizes and has precluded large-scale longitudinal studies [148]. Some studies have used non-endoscopic sampling techniques, including the Esophageal String Test [146], Cytosponge [149], and inflatable balloons [150]. While these non-endoscopic samples yielded microbial profiles similar to endoscopic biopsies and brushings, the non-endoscopic approaches do not sample exclusively from the esophagus and include microorganisms from other parts of the upper gastrointestinal (UGI) tract, including the oral cavity and stomach [146,149,150]. Esophageal brushings have been shown to yield greater bacterial DNA and lower human DNA than biopsies [151], which is important to consider given the low microbial biomass in the esophagus. In addition to the lower cost and less risk associated with the procedure, non-endoscopic techniques such as the Cytosponge have the added benefit of obtaining a greater amount of microbial DNA compared with both endoscopic biopsies and brushings (Cytosponge yielded greater than 10-times more microbial DNA than did biopsies/brushings) [149]. Non-endoscopic sampling approaches may also provide a more comprehensive view of the esophageal microbiome than would be obtained with a single 3 mm mucosal biopsy sample, given the larger surface area that is sampled with non-endoscopic techniques [146]. Culture-independent studies of the esophageal microbiome are currently mostly restricted to 16S rRNA gene sequencing, and more detailed profiling using metagenomic sequencing is hampered since the majority of studies have used endoscopic samples of the esophagus with limited microbial biomass [148]. 

### 6.2. Factors That Impact the Esophageal Microbiome

The esophageal microbiome is relatively understudied, but several factors have been found to impact the composition of the bacterial community in the esophagus. Age was shown to be associated with the esophageal microbial composition, with age being positively and inversely correlated with abundances of *Streptococcus* spp. and *Prevotella* spp., respectively [152]. While comprehensive studies of the relationship between diet and the esophageal microbiome are lacking, one study found that dietary fiber, but not dietary fat, was associated with differences in the esophageal microbiome [153]. In this study, increasing fiber intake was significantly correlated with increases in the relative abundance of Firmicutes (*p* = 0.04) and decreases in Gram-negative bacteria overall (*p* = 0.03), whereas low fiber intake was associated with increases in several Gram-negative bacteria. This study also found that increasing BMI was independently associated with decreasing relative abundance of Firmicutes (*p* = 0.03) and increasing Gram-negative bacteria (*p* = 0.007), which was the opposite of associations seen for fiber intake. In another study of balloon cytology samples collected from healthy Chinese adults, BMI was associated with differences in the UGI microbial composition (beta diversity), which further suggest that obesity may impact the esophageal microbiome [154]. The UGI microbiome has also been shown to be influenced by oral health [155]. Yu et al. found that microbial richness in the UGI tract was positively correlated with poor periodontal health, whereas it was negatively correlated with poor dental health (as assessed by missing teeth, tooth decay, and DMFT score). In addition, the presence of potentially pathogenic genera *Parvimonas* and *Porphyromonas* was associated with poor periodontal health (*p* < 0.001), and subjects with *Veillonellaceae [G-1]* had a significantly higher DMFT score (*p* < 0.001). Although few in number, these studies show that lifestyle and environmental exposures may modify the esophageal microbiome. 

GERD, which is one of the main risk factors for EAC, has also been shown to impact the esophageal microbiome. Similar to the normal esophagus, Firmicutes and Proteobacteria are the predominant phyla in the esophageal microbiome of GERD patients [156,157,158]. However, several studies have found that the esophageal microbiome in GERD becomes shifted towards an increase in Gram-negative bacteria and a decrease in Gram-positive bacteria (mainly *Streptococcus*) [145,152,159,160] compared with the normal esophagus. Some Gram-negative genera found to be enriched in GERD compared with normal esophagus include *Veillonella*, *Prevotella*, *Neisseria*, *Campylobacter*, *Leptotrichia*, and *Fusobacterium* [145,152,157,159,160]. One study found that the esophageal microbiome of patients with non-erosive reflux disease (NERD), a milder form of GERD, was distinct from those with erosive reflux esophagitis (RE) and normal esophagus [160]. The NERD microbiome was characterized by shifts towards an increase in Bacteroidetes and Proteobacteria and a decrease in Fusobacteria and Actinobacteria, along with an increase in the genus *Dorea* of the Firmicutes phylum compared with controls and RE patients. These existing studies were limited to small sample sizes and cross-sectional designs, so additional studies are required to better understand microbial changes associated with the transition from the normal esophagus to GERD.

Acid suppressants such as proton pump inhibitors (PPIs), which are commonly used to treat reflux-related esophageal disorders, are suspected to have important impacts on the esophageal microbiome, but it remains unclear whether they influence the EAC cascade [161]. PPIs are thought to alter the esophageal and gastric microbiome by increasing gastric pH and decreasing distal esophageal mucosal acid exposure [161] and/or by directly targeting proton pumps of certain bacteria that contain P-type ATPase enzymes (e.g., *Streptococcus pneumoniae* and *H. pylori*) [162]. In a study examining esophageal biopsies and gastric fluid before and after 8 weeks of PPI treatment in eight patients with GERD/BE/esophagitis, Amir et al. found that both the esophageal and gastric microbiome were significantly altered with PPI treatment [158]. After PPI treatment, relative abundances of *Comamonadaceae* were decreased, whereas three families from the phylum Clostridia (i.e., *Clostridiaceae*, *Lachnospiraceae*, and an unclassified family), *Microccocaceae*, *Actinomycetaceae*, and unclassified families from the orders Lactobacillales and Gemellales (both from the phylum Firmicutes) were increased in the esophagus. Snider et al. found that PPI users (6/16 patients without BE and 29/29 BE patients) had higher and lower relative abundances of *Streptococcus* (*p* = 0.03) and Gram-negative bacteria (*p* = 0.05) compared with nonusers (10 patients without BE) [163]. In a study by Deshpande et al., there was no effect of PPI use among individuals with a normal esophagus (based on both alpha and beta diversity measures), whereas PPI use had a small effect on the microbial composition of GERD patients [152]. Another study found that there was no effect of PPI use on alpha and beta diversity of the esophageal microbiome in GERD and BE patients, but PPI use was associated with an increase in Firmicutes and a decrease in Bacteroidetes and Proteobacteria [160]. Tasnim et al. used a custom quantitative PCR array to measure select microorganisms in biopsies from the distal esophagus to examine the effect of PPI use on the esophageal microbiome in 58 GERD patients (including 26 with BE), among which 52 were PPI users and 6 were nonusers [164]. The authors found that PPI users had significantly higher levels of *Actinomyces* (*p* < 0.01) compared with nonusers, but there was no difference in the other organisms that were measured. In addition, the dose (20–80 mg) and duration (1–13 years) of PPI use was not associated with the abundance of any of the organisms. Without longitudinal studies of larger cohorts, it is difficult to determine exactly how PPIs alter the esophageal microbiome and whether this impacts EC risk. Many existing esophageal microbiome studies have treated PPI use differently, with some studies excluding subjects using PPIs within a certain amount of time prior to the study, and others not clearly indicating the use of PPIs or other drugs that may affect the microbiome composition [141,165]. Given that PPIs may impact the esophageal microbiome, future studies should account for the use of these medications. 

## 7. Esophageal Microbiome and EC

### 7.1. Esophageal Microbiome and ESCC

Few studies have examined the esophageal microbiome in ESCC, all of which have been cross-sectional analyses (Table 2). Yu et al. compared the UGI microbiome of 142 subjects with ESD (the precursor lesion for ESCC) and 191 control subjects without ESD and found that the presence of dysplasia was associated with lower microbial richness [150]. Another study by Shao et al. characterized microbial communities of paired tumor and nontumor samples from 67 ESCC patients and 36 patients with gastric cardia adenocarcinoma, which commonly co-occurs at high rates in many of the same geographic regions with high incidence of ESCC [166]. Although there was no difference in alpha diversity between the ESCC tumor and nontumor samples, significant clustering was detected in beta diversity analysis using the weighted UniFrac distance matrix (*p* = 0.015) but not for unweighted UnifFrac. This suggests that similar taxa were present in ESCC tumor and nontumor samples, but there were differences in the relative abundances of the taxa. When looking at taxa-specific relative abundances, ESCC tumor samples contained higher relative abundance of *Fusobacterium* (3.2% vs. 1.3%) and lower relative abundance of *Streptococcus* (12.0% vs. 30.2%) compared with the paired nontumor samples. There was a positive association between the relative abundance of *Fusobacterium* and increasing ESCC tumor stage, although this was not statistically significant (*p* = 0.29). On the other hand, the relative abundance of *Streptococcus* significantly decreased with an increase in ESCC tumor stage (*p* = 0.030). The increased level of *Fusobacterium* in ESCC was also found in the study by Li et al. that examined the esophageal microbiome of patients with ESCC (*n* = 17), esophagogastric junction cancer (*n* = 11), along with patients after esophagectomy (*n* = 15) and healthy control subjects (*n* = 16) [167]. In this study, ESCC subjects had lower microbial richness and evenness (alpha diversity measured by the Sobs (*p* = 0.008) and Shannon (*p* = 0.017) indices) compared with control subjects, and ESCC samples were enriched in *Streptococcus*, *Lactobacillus*, *Prevotella*, and *Fusobacterium*. Significant differences were found in the microbial compositions of ESCC and control subjects (based on beta diversity analysis using unweighted UniFrac), and the key taxa contributing to the changes in the esophageal microbiome of ESCC patients were identified to be *Clostridiales*, *Pseudomonas*, and *Selenomonadales*. In a study by Nasrollahzadeh et al., the gastric corpus microbiome was compared between cases with early ESCC and ESD (*n* = 37) and a control group consisting of subjects with mid-esophagus esophagitis (diseased controls, *n* = 17) and histologically normal esophagus (healthy controls, *n* = 37) [168]. No significant differences were detected in alpha diversity (measured by the Chao1 index) between cases and controls, but there were some differences in microbial compositions between matched pairs of cases and controls as assessed by weighted and unweighted UniFrac distance measures. Order-level analyses of individual taxa showed that Clostridiales (adj *p* = 0.011) and Erysipelotrichales (adj *p* = 0.011) were significantly higher in abundance among cases compared with healthy controls. 

There have also been some studies that have performed targeted investigations of associations between specific bacterial infections and ESCC. The periodontal pathogen *P. gingivalis* has been shown to be more present in cancerous esophageal tissues of ESCC patients, whereas it was undetected in esophageal mucosa from normal controls in a cross-sectional analysis using immunohistochemistry [169]. The presence of *P. gingivalis* in ESCC patients has also been correlated with ESCC stage, poor prognosis, and reduced chemotherapy efficacy [170,171,172]. Similarly, *F. nucleatum* DNA was found to be present at higher levels in ESCC cancer tissues than in paired adjacent nontumor tissues as measured by qPCR [173], and high levels of *F. nucleatum* in ESCC tumor tissues have been correlated with a poor prognosis and lower chemotherapeutic response [174,175]. These studies suggest that specific bacteria such as *P. gingivalis* and *F. nucleatum* may potentially be useful as prognostic markers, but this does not necessarily indicate that these bacteria play prominent roles in the etiology of ESCC. Furthermore, it is unclear whether eradication of specific bacteria is effective in improving disease outcomes. 

Studies of the esophageal microbiome and ESCC have been limited to extremely small sample sizes and restricted to cross-sectional analyses, primarily due to difficulties in obtaining large numbers of samples via endoscopy. Larger studies, ideally with prospective designs, are needed, and non-endoscopic sampling techniques may be better suited for this purpose. Spurious results cannot be ruled out with such small sample sizes, and the findings from existing studies will need to be replicated in future studies, such as the possible involvement of *F. nucleatum* in ESCC. 

### 7.2. Esophageal Microbiome and BE 

A number of cross-sectional analyses have found differences in the esophageal microbiome of the normal esophagus and BE, the precursor lesion for EAC (Table 2). Similar to GERD, the esophageal microbiome of BE is increasingly dominated by Gram-negative bacteria, which is in contrast to the mostly Gram-positive microbiome dominated by the genus *Streptococcus* in the normal esophagus [145,152,157,159,160,176]. In a comprehensive analysis of the esophageal microbiome by Deshpande et al. using 16S/18S rRNA sequencing as well as shotgun sequencing [152], *Campylobacter*, along with other Gram-negative genera including *Leptotrichia*, *Fusobacterium*, *Rothia*, and *Capnocytophaga*, were also found to be enriched in subjects with esophageal diseases at the early stages of the EAC cascade (*n* = 29 with GERD, 7 with glandular mucosa, and 5 with BE) compared with control subjects (*n* = 59) with histologically normal esophagus. Other studies have also detected high levels of the Gram-negative genus *Campylobacter* in BE patients [145,152,159,163,176]. Macfarlane et al. detected *Campylobacter concisus* and *Campylobacter rectus* in more than half of BE patients (4 out 7, 57%) but these species were not present in control subjects (*n* = 7) with UGI symptoms but without BE [176]. Blackett et al. also observed marked higher levels of *C. concisus* (in both prevalence and counts) in subjects with GERD (*n* = 37) and BE (*n* = 45) relative to control patients with no esophageal diseases or any reflux symptoms [159]. In agreement with the study by Deshpande et al. [152], additional studies have found increased levels of *Leptotrichia* [160,177] and *Fusobacterium* [157,176] (both Gram-negative genera of the phylum Fusobacteria) compared with the normal esophagus. *Veillonella* is another subdominant Gram-negative taxa that has been shown to be enriched in BE compared with the normal esophagus [145,157,176,177]. Repeated exposure to gastric acid and bile salts in refluxate are thought to contribute to inflammation and injury of the esophageal squamous epithelium, and these changes in the local environment are likely to be contributing to the alterations in the microbiome of BE compared with the normal squamous esophagus [158]. One study has shown that the gastric refluxate microbiome composition was modified in patients with an abnormal esophagus (*n* = 11 esophagitis and 5 BE) compared with individuals with heartburn and normal esophagus (*n* = 14), as demonstrated by the separation between the two groups using unweighted UniFrac beta diversity analysis (ANOSIM R = 0.24, *p* < 0.0006) [158]. This study also identified that *Enterobacteriaceae* (specifically genus *Esherichia*) were enriched in gastric fluid samples of patients with esophagitis and BE compared with normal esophagus (*p* = 0.009). Members of the Gram-negative *Enterobacteriaceae* have been implicated in inflammatory bowel disease and irritable bowel syndrome, and it is possible that they may also contribute to inflammation in the esophagus [158]. 

Some studies suggest the ratio of *Streptococcus* and *Prevotella* may be an important indicator of esophageal disease. A co-exclusion relationship between the two dominant genera *Streptococcus* and *Prevotella* was observed in the esophageal microbiome of GERD, glandular mucosa, and BE in the study by Deshpande et al., which showed that the ratio of the relative abundances of the two genera defined functionally distinct esophageal community types [152]. Interestingly, the esophageal community type dominated by *Prevotella* was enriched for lipopolysaccharide biosynthesis pathways. It is hypothesized that bacterial antigens specific to Gram-negative bacteria, including lipopolysaccharide, may promote tissue inflammation and may also increase reflux by relaxing the lower esophageal sphincter and delaying gastric emptying, thereby promoting GERD and BE [52]. Similarly, Lopetuso et al. found that there was a reduction in the relative abundance of Gram-positive *Streptococcus* and a corresponding increase in Gram-negative *Prevotella* when comparing the esophageal microbiome of normal esophagus (*n* = 10) with BE (*n* = 10) using esophageal biopsies [177]. Furthermore, another study looking at biopsy and brush samples collected from the squamous esophagus, BE, stomach corpus, and stomach antrum from 12 BE patients found a statistically significant inverse relationship between the *Streptococcus*:*Prevotella* ratio in BE and hiatal hernia length (*r*^2^ = 0.60, *p* = 0.001), which is a known risk factor for both BE and EAC [151]. 

In addition to changes in the abundance of individual bacteria, it appears there may also be polymicrobial community-level changes in BE, along with alterations in the microbial functional profiles. Networks of strongly correlated bacteria have been detected in the esophageal microbiome, and markedly denser microbial networks were observed with the progression of disease from normal esophagus to GERD and BE [152,177]. Using shotgun sequencing data to determine functional changes in the esophageal microbiome, Deshpande et al. [152] found that lactic acid production pathways, including homolactic fermentation and heterolactic fermentation, were enriched in GERD and BE, respectively, compared with subjects with a normal esophagus. These studies suggest that rather than focusing on a specific pathogen, it may be necessary to look at community-level changes and alterations in the functional activities of the microbial community to better understand the etiology of BE. 

The microbiome of metaplastic mucosa may be different from unaffected areas of the esophagus in BE patients. In a comparison of metaplastic and adjacent healthy tissue from 10 BE patients, there was no difference in alpha diversity, but metaplastic tissue showed lower relative abundances of several taxa, including Bacteroidetes (*p* = 0.049) and TM7 (*p* = 0.002) at the phylum level and *Prevotella* (*p* = 0.027), *Fusobacterium* (*p* = 0.04), *Campylobacter* (*p* = 0.008), and *Selenomonas* (*p* = 0.006) at the genus level [177]. In another study of 12 BE patients, esophageal biopsies taken from the proximal, mid, and distal esophagus, along with BE mucosa, showed differences in the relative abundances of various taxa among these sites [147]. In this study, the Gram-negative *Haemophilus* was found to be abundant in BE tissue but relatively absent elsewhere in the esophagus, and BE tissue was dominated by a larger percentage of Gram-negative organisms compared with other sites in the esophagus. With non-endoscopic sampling techniques such as the Cytosponge, the fraction of the microbiome sampled from BE may be diluted by bacteria sampled from other parts of the UGI tract [149], and so esophageal biopsies may be required to compare metaplastic and healthy esophageal mucosa in BE patients. However, non-endoscopic approaches, including the Cytosponge, may still be useful for detecting differences in the overall esophageal microbiome of BE patients and individuals with a normal esophagus [149]. 

Although existing studies of the BE microbiome have been limited to cross-sectional analyses and small sample sizes, there have been some consistent findings, such as the possible involvement of Gram-negative bacteria and changes in the ratio of the two major genera, *Streptococcus* and *Prevotella*. However, the cross-sectional designs of these studies preclude inferences about temporality, and so it remains unclear whether changes in the microbiome result from BE or vice versa. Prospective studies with larger sample sizes are required to confirm whether Gram-negative bacteria and changes in the *Streptococcus*:*Prevotella* ratio are associated with the etiology of BE and how these alterations relate to the transition from a normal esophagus to BE. 

### 7.3. Esophageal Microbiome and EAC

The esophageal microbiome in EAC is not well characterized and has only been studied in a few small-scale cross-sectional studies (Table 2). Although there seems to be no difference in alpha diversity between the normal esophagus and BE, there is some evidence of a decrease in alpha diversity in EAC [149,163]. Beta diversity analyses have shown that the microbial community structure significantly differs between EAC and the normal esophagus [149,160,177] and also between EAC and BE [177]. However, unlike the BE microbiome, there does not seem to be a clear, consistent pattern of change in the microbial composition of EAC compared with a normal esophagus. In a comparison of normal (*n* = 10), BE (*n* = 10), and EAC (*n* = 6) mucosa by Lopetuso et al. [177], the decrease in the *Streptococcus*:*Prevotella* ratio seen in BE compared with the normal esophagus was more marked between EAC and the normal esophagus. In contrast, some studies have noted an enrichment of lactic acid bacteria, including *Streptococcus*, in the EAC microbiome [149,160]. In a comparison of esophageal biopsies from patients with non-dysplastic BE (*n* = 17), EAC (*n* = 15), and controls with a normal esophagus (*n* = 16), Elliott et al. showed that *Lactobacillus fermentum* was enriched in EAC patients compared with control subjects and those with BE and that acid-tolerant Lactobacillales (*Lactobacillus* spp. and *Streptococcus* spp.) were predominant in 7 of the 15 (47%) EAC patients [149]. However, six of the seven EAC patients with high levels of Lactobacillales were taking antacid drugs (such as PPIs), which may have impacted the esophageal microbial composition of these subjects. In another study including 6 EAC patients and 16 control subjects with no esophageal diseases or GERD symptoms, the EAC microbiome consisted of a shift towards the phylum Firmicutes, with high abundances of lactic acid producing bacteria including *Staphylococcus*, *Bifidobacterium*, *Streptococcus*, and *Lactobacillus*, compared with the normal esophagus [160]. Only one of the six EAC patients was taking PPIs in this study. Additional studies are needed to determine whether lactic acid bacteria are enriched in EAC, or whether this is related to taking acid suppressants such as PPIs. 

Several other bacteria have been shown to be differentially abundant in EAC compared with the normal esophagus and BE. In the study by Blackett et al., *Campylobacter concisus* was enriched in patients with GERD and BE compared with control subjects with normal esophagus, but *C. concisus* was relatively absent in EAC patients (only present in 3 out of 30 EAC subjects), which suggests this species may be associated with refluxate in the esophagus (refluxate is reduced in most cancers) [159]. In the aforementioned study by Elliott et al. [149], several Gram-negative genera, including *Campylobacter*, *Veillonella*, and *Megasohaera*, along with the Gram-positive genera *Granulicatella*, *Atopobium*, *Actinomyces*, and *Solobacterium*, were found to be decreased in EAC patients relative to controls with normal esophagus and BE patients. Snider et al. found that the microbiome of esophageal brushings collected from the combined group of patients with high-grade dysplasia (*n* = 5) and EAC (*n* = 4) had decreased Firmicutes and increased Proteobacteria compared with the group of patients with BE without dysplasia (*n* = 14) and low-grade dysplasia (*n* = 6) [163]. Similar to Elliott et al. [149], *Veillonella* was noticeably reduced in patients with high-grade dysplasia/EAC compared with patients with non-dysplastic BE/low-grade dysplasia in the study by Snider et al. In contrast, EAC patients were found to have higher relative abundances of *Veillonella* compared with controls and patients with BE in the study by Lopetuso et al. [177], which suggests there may be inconsistencies across studies in the findings related to this low-abundance bacterium, possibly due to measurement error or due to spurious findings caused by the small sample sizes. Lopetuso et al. also found that *Leptotrichia* was a distinguishing taxon for EAC, despite being one of the less abundant species of the normal esophageal microbiome [177]. In the study by Snider et al., patients with high-grade dysplasia/EAC were also characterized by an increase in *Enterobacteriaceae* and *Akkermansia muciniphila* [163]. Interestingly, Snider et al. also previously showed that *Enterobacteriaceae* was increased in saliva of patients with high-grade dysplasia/EAC [140], suggesting there may be correlations between the salivary and esophageal microbiome. 

Very little is understood about the esophageal microbiome in EAC, with existing studies being restricted to small cross-sectional analyses, mostly consisting of fewer than 10 EAC patients. Investigation of the EAC microbiome is still early, and replication of findings in larger studies will be needed to identify consistent patterns of change across the normal esophagus, BE, and EAC. It is unclear at this time whether alterations in the esophageal microbiome are directly involved in the etiology of EAC. Future studies need to account for the use of acid suppressants and how this may be altering the EAC microbiome. 

## 8. Conclusions and Future Directions

To date, studies investigating the relationship between the microbiome and EC are limited in number, and it cannot be determined whether there is a causal association at this time. However, existing studies included in this review provide enough suggestive evidence of alterations in the oral and esophageal microbiome related to EC. The exact nature of the association between the microbiome and EC is yet to be determined, underlining the need for additional studies in the future. For the oral microbiome and EC, it is unclear whether specific oral pathogens related to periodontal disease and dental caries directly contribute to risk or whether community shifts resulting from these poor oral health conditions impact EC risk. Questions remain about how risk factors such as poor oral health and GERD alter the esophageal microbiome and how this may contribute to EC. In particular, additional studies investigating the effects of poor oral health conditions on the oral microbiome, and downstream effects on the esophageal microbiome, are warranted to better understand the mechanism for the association between oral health and EC. This may inform clinical practices of the importance of oral hygiene practices and prophylactic dental treatments in reducing the burden of EC. 

This review article has several strengths and limitations. One strength is that we paid careful attention to the different histologic subtypes of EC. We explored the relationship between the microbiome and esophageal cancer from multiple aspects, separately linking them back to known epidemiological risk factors for ESCC and EAC. Another strength is that in addition to the esophageal microbiome, we examined the oral microbiome, which has a clear connection to oral health, a suspected risk factor for EC (particularly ESCC). However, we did not explore the connection between EC and the microbiome of other body sites, such as the gut microbiome. We also focused only on bacterial communities and did not study the involvement of other microorganisms (e.g., fungi, viruses). This review is also limited by the small number of studies that were included, and the cross-sectional designs and low power in many of the existing studies did not allow us to draw strong conclusions about the relationship between the microbiome and EC. 

Epidemiologic investigation of associations between the human microbiome and EC is still in the early stages. Existing studies of the oral and esophageal microbiome and EC have largely been restricted to cross-sectional designs that limit etiologic inferences. Most EC microbiome studies do not clearly specify whether they are assessing disease effects or whether they are aiming to identify etiologic exposures, which is an important distinction to make in these investigations. Thus, temporality remains a key question, which will require prospective studies in which microbial exposures are assessed prior to the onset of disease to directly examine whether microbiome changes are causal agents of EC or whether EC causes changes in the microbiome. 

Another major limitation of the current literature on the microbiome and EC is that many of the studies have very limited sample sizes. In the existing oral and esophageal microbiome studies of EC, it was not uncommon for studies to have sample sizes of fewer than 50, and most were under 100, particularly for studies of the esophageal microbiome. These small studies may not be powered to detect meaningful differences between disease states, and some of the findings may have been spurious. For example, in a previous study assessing the temporal variability of the oral microbiome, it was estimated that for oral samples collected using Scope mouthwash, given an intraclass correlation coefficient (an index used to assess the reliability of a microbiome measurement—numbers closer to 0 and 1 indicate poor and good reliability, respectively) of 0.47 for the relative abundance of Firmicutes and a single specimen collection per subject, 1401 cases and 1401 controls would be required to detect an OR of 1.50 at the significance level of 0.05 [178]. To detect a larger effect size, with an OR of 3.50 (which is rather uncommon in such studies), the number of required subjects becomes 141 cases and 141 controls. The required sample sizes decrease for microbial measures with smaller temporal variability (i.e., higher intraclass correlation coefficients), larger effect sizes, and with repeated sampling from each study participant [178]. However, samples sizes of at least 200 (100 cases and 100 controls) may be necessary to detect robust associations. Furthermore, replication in independent studies is critical to distinguish true associations from spurious ones. Another important consideration in high-dimensional microbiome data is that corrections for multiple comparisons are needed to prevent false positives when testing for taxa-specific associations with disease. In addition to the use of reproducible experimental protocols to generate microbiome data [179], the uncertainty of results (particularly from small studies) should be recognized, and statistical analyses must be performed carefully to prevent the field from drowning in irreproducible but “significant” findings [180].

There is a clear need for prospective studies in large cohorts to better understand how the microbiome is involved in the etiology of EC. Nested case-control studies with prospective samples provide an efficient means of assessing etiologic factors in the microbiome associated with EC risk, and because samples are collected prior to disease onset, reverse causation is less likely to occur. Large-scale studies of the esophageal microbiome may be challenging due to the difficulty of collecting esophageal samples, but this may be more feasible with non-endoscopic collection methods or in situations where endoscopic screening is widespread. Large prospective studies of the oral microbiome and EC may be easier to conduct compared with esophageal microbiome studies since oral samples for microbial analysis can be obtained with home collection kits using mouthwash. In fact, buccal cells obtained using mouthwash in existing cohorts for human genomic studies can also be used to study the oral microbiome [181]. Future investigations of the microbiome and EC should also include assessments of oral health measures, particularly for studying the relationship between the oral microbiome and ESCC. 

Although specific microorganisms may be able to drive carcinogenesis, as exemplified by *H. pylori* and gastric cancer, it may be more likely that the etiology of EC involves a polymicrobial process rather than being caused by infection with a single specific pathogen. For example, studies specifically focusing on single bacteria, including *Fusobacterium nucleatum* and *Porphyromonas gingivalis*, have found that high levels of these bacteria in ESCC tumor tissues were associated with a poor prognosis/survival, but these associations should be verified in untargeted studies looking at the microbial community as a whole to see if these specific bacteria are really the driving agents for a poor prognosis. Given that some studies have identified networks of bacteria present in diseased states, it is possible that pathogenic bacteria are acting in consortia with other microorganisms to drive community-level shifts in the microbiome towards a diseased state. Due to the considerable overlap in the functional potential of bacterial species, some studies have suggested that a better understanding of disease etiology may be obtained by studying the functional activities of microbes, as opposed to attempting to identify a specific microbial composition related to disease [119,182]. Functional analyses are warranted in the future, using techniques in metabolomics, proteomics, metagenomics, and metatranscriptomics to study changes in functional profiles of the microbiome associated with EC. Finally, while this review focused solely on bacteria, other members of the microbiome, including fungi, archaea, protozoa, viruses, and the interplay between these microbes, are also likely to be involved in the etiology of EC. 

## Figures and Tables

**Table 1:** Studies of the oral microbiome and esophageal cancer.

							Main Findings		
Author (Year)	Study Design	Country	Cases (N)	Controls (N)	Sample Type	Method	Alpha Diversity	Beta Diversity	Differentially Abundant Taxa
Chen 2015, [135]	Cross-sectional case-control	China	87 ESCC, 63 ESD	85	Saliva	16S rRNA, NGS	Lower alpha diversity in ESCC compared with ESD and controls	Separation between ESCC cases and controls, with ESD in between the two groups	*Prevotella*, *Streptococcus*, and *Porphyromonas* were higher and most other genera were lower in ESCC cases compared with controls and ESD.
Wang 2019, [136]	Cross-sectional case-control	China	20 ESCC	21	Saliva	16S rRNA, NGS	No difference	Some separation along third principal coordinate, but overall, no clear separation	Some differences in *Actinomyces*, *Atopobium*, *Fusobacterium*, *Porphyromonas*, but none significant after multiple testing correction.
Zhao 2020, [137]	Cross-sectional case-control	China	33 ESCC, 6 EAC	51	Saliva	16S rRNA, NGS	No difference	Separation between EC cases (combined) and controls	Notable differences in *Firmicutes*, *Negativicutes*, *Selenomonadales*, *Prevotellaceae*, *Prevotella*, *Veillonellaceae*, *Proteobacteria*, *Betaproteobacteria*, *Neisseriales*, *Neisseriaceae*, and *Neisseria* between EC cases (combined) and controls.
Liu 2020, [138]	Prospective case-control	China	84 esophageal lesions of severe squamous dysplasia and above (severe squamous dysplasia, carcinoma in situ, ESCC)	168	Oral swab	16S rRNA, NGS	Higher alpha diversity in cases	No difference	Identified 11 species that may be predictive of risk: *Actinomyces odontolyticus*, *A. viscosus*, *Dialister invisus*, *Fusobacterium mortiferum*, *F. nucleatum*, *Lachnoanaerobaculum umeaense*, *Leptotrichia hofstadii*, *Prevotella baroniae*, *P. melaninogenica*, *P. shahii, Rothia dentocariosa*.
Peters 2017, [139]	Prospective case-control	US	25 ESCC, 81 EAC	210	Oral wash	16S rRNA, NGS	No difference	No difference	*Porphyromonas gingivalis* was modestly associated with increased ESCC risk. *Tannerella forsythia* was associated with increased EAC risk. Depletion of *Neisseria* and *Streptococcus pneumoniae* was associated with decreased EAC risk.
Snider 2018, [140]	Cross-sectional case-control	US	32 BE (16 BE, 6 low grade dysplasia, 5 high grade dysplasia, 5 EAC)	17	Saliva	16S rRNA, NGS	No difference	Separation between BE (combined) and controls	*Streptococcus*, *Veillonella*, and *Enterobacteriaceae* higher in BE (combined), and numerous taxa were higher in controls (e.g., *Neisseria*, *Lautropia*, *Corynebacterium*)

EAC, esophageal adenocarcinoma; ESCC, esophageal squamous cell carcinoma; ESD, esophageal squamous dysplasia; NGS, next-generation sequencing.

**Table 2:** Studies of the esophageal microbiome and esophageal cancer.

							Main Findings		
Author (Year)	Study Design	Country	Cases (N)	Controls (N)	Sample Type	Method	Alpha Diversity	Beta Diversity	Differentially Abundant Taxa
Yu 2014, [150]	Cross-sectional case-control	China	142 ESD	191	Balloon	HOMIM	Microbial richness lower in ESD	First principal component associated with ESD status	None.
Shao 2019, [166]	Cross-sectional	China	67 ESCC, 36 gastric cardia adenocarinoma	–	Biopsy	16S rRNA, NGS	No difference between ESCC tumor and paired nontumor samples	Separation between ESCC tumor and nontumor samples	ESCC tumor samples had higher *Fusobacterium* and lower *Streptococcus* compared with paired nontumor samples. Inverse association between *Streptococcus* and ESCC tumor stage.
Li 2020, [167]	Cross-sectional case-control	China	17 ESCC, 11 esophagogastric junction cancer, 15 post-esophagectomy	16	Biopsy	16S rRNA, NGS	Overall lower alpha diversity in all case groups compared with controls	Separation between ESCC and controls	*Streptococcus*, *Lactobacillus*, *Prevotella*, and *Fusobacterium* enriched in ESCC compared with controls. *Fusobacteria* was higher and *Actinobacteria* was lower in ESCC compared with controls. Key taxa distinguishing ESCC patients identified to be *Clostridiales*, *Pseudomonas*, *and Selenomonadales.*
Nasrollahzadeh 2015, [168]	Cross-sectional case-control	Iran	37 early ESCC and ESD	17 diseased controls (mid-esophageal esophagitis), 37 healthy controls	Biopsy	16S rRNA, NGS	No difference	Some differences in principal coordinates between cases and controls	Clostridiales and Erysipelotrichales higher in cases compared with healthy controls.
Macfarlane 2007, [176]	Cross-sectional case-control	UK	7 BE	7	Biopsy, aspirate	Culture	–	–	*Campylobacter* (*C. concisus* and *C. rectus*) in most BE patients but in none of the controls.
Yang 2009, [145]	Cross-sectional case-control	US	12 esophagitis, 10 BE	12	Biopsy	16S rRNA gene clones	–	Separation between cases (combined) and controls	*Streptococcus* predominant in controls. Increased Gram-negative bacteria in BE and esophagitis.
Blackett 2013, [159]	Cross-sectional case-control	UK	37 GERD, 45 BE, 30 EAC	39	Biopsy	Culture/qPCR	–	–	Higher *Campylobacter* (*C. concisus* was the dominant species) in GERD and BE compared with controls but not with EAC.
Liu 2013, [157]	Cross-sectional case-control	Japan	6 esophagitis, 6 BE	6	Biopsy	16S rRNA gene clones	–	–	*Streptococcus* predominant in controls and reflux esophagitis. *Veillonella*, *Neisseria*, and *Fusobacterium* present in reflux esophagitis and BE but not controls.
Amir 2014, [158]	Cross-sectional case-control	Israel	13 GERD with esophagitis, 6 BE	15 GERD without esophagitis	Biopsy, gastric fluid	16S rRNA, NGS	–	No separation between esophageal biopsies from esophagitis, BE, and controls; separation between gastric fluid samples from controls and abnormal esophagus (esophagitis and BE combined)	No differential taxa for esophageal biopsies from abnormal esophagus (esophagitis and BE) and controls. Higher *Enterobacteriaceae* (specifically the genus *Escherichia*) and *Methylobacteriaceae* in gastric fluid of esophagitis and BE compared with controls. *Pasteurellaceae* and *Porphymonodaceae* higher in controls compared with esophagitis and BE.
Gall 2015, [151]	Cross-sectional	US	12 BE	–	Biopsy, brushing	16S rRNA, NGS	–	–	Inverse correlation between *Streptococcus*:*Prevotella* ratio and hiatal hernia length (risk factor for BE and EAC) in the stomach corpus, stomach antrum, and BE.
Elliott 2017, [149]	Cross-sectional case-control	UK	24 non-dysplastic BE, 23 dysplastic BE, 19 EAC	20	Biopsy, brushing, Cytosponge	16S rRNA, NGS/qPCR	Alpha diversity lower in EAC compared to controls	Separation between EAC and controls	BE had higher Proteobacteria compared with controls and EAC. *Campylobacter*, *Veillonella*, *Megasohaera*, *Granulicatella*, *Atopobium*, *Actinomyces*, and *Solobacterium* lower in EAC compared with BE and controls. *Lactobacillus fermentum* enriched in EAC compared with BE and controls, and Lactobacillales (*Lactobacillus* spp. and *Streptococcus* spp.) predominant in EAC.
Deshpande 2018, [152]	Cross-sectional case-control	Australia	29 GERD, 7 glandular mucosa, 5 BE, 1 EAC, 1 eosinophilic esophagitis	59	Biopsy, brushing	16S/18S rRNA, NGS/shotgun	No difference by disease status	No difference by disease status	Gram-negative bacteria (e.g., *Leptotrichia*, *Fusobacterium*, *Rothia*, *Campylobacter*, *Capnocytophaga*) enriched in GERD, glandular mucosa, and BE. Microbial lactic acid production was increased in GERD and BE. *Streptococcus*:*Prevotella* ratio defined functionally distinct esophageal communities.
Snider 2019, [163]	Cross-sectional case-control	US	14 BE without dysplasia, 6 low grade dysplasia, 5 high grade dysplasia, 4 EAC	16	Brushing	16S rRNA, NGS	No difference between BE cases (combined) and controls; among BE cases, EAC had decreased Simpson index	No difference	Combined group of high-grade dysplasia and EAC had decreased Firmicutes and increased Proteobacteria compared with the group of BE without dysplasia and low-grade dysplasia. Group of high-grade dysplasia and EAC had increased *Enterobacteriaceae* and *Akkermansia muciniphila* and reduced *Veillonella.*
Okereke 2019, [147]	Cross-sectional	–	12 BE	–	Biopsy	qPCR	–	–	*Haemophilus* abundant in BE tissue but relatively absent elsewhere in the esophagus. BE tissue dominated by a larger percentage of Gram-negative organisms compared with other sites in the esophagus.
Lopetuso 2020, [177]	Cross-sectional case-control	Italy	10 BE, 6 EAC	10	Biopsy	16S rRNA, NGS	Higher alpha diversity in BE and EAC compared with controls, but not statistically significant	Separation between EAC and controls and between EAC and BE	Progressive reduction in *Streptococcus* and corresponding increase in *Prevotella* in BE and EAC. *Leptotrichia* was a distinguishing taxon for EAC.
Zhou 2020, [160]	Cross-sectional case-control	Australia	11 NERD, 20 RE, 17 BE, 6 EAC	16	Biopsy, brushing	16S rRNA, NGS	Lower Chao1 in NERD compared with controls and RE; no difference in Shannon index	Some differences between EAC and controls	Controls had higher Firmicutes and Actinobacteria compared with other groups. NERD had higher Proteobacteria and Bacteroidetes and lower Fusobacteria and Actinobacteria, along with decreases in several Firmicutes genera including *Dorea*. RE and BE had lower Firmicutes and increased Gram-negative Fusobacteria and Proteobacteria compared with controls. EAC shifted towards Firmicutes (mainly *Staphylococcus aureus*, *Streptococcus infantis*, *Moryella* sp. and *Lactobacillus salivarius*) and Proteobacteria, while shifting away from Actiobacteria (*Rothia mucilaginosa*) compared with controls.

– absent or not applicable. BE, Barrett’s esophagus; EAC, esophageal adenocarcinoma; ESCC, esophageal squamous cell carcinoma; ESD, esophageal squamous dysplasia; GERD, gastroesophageal reflux disease; HOMIM, Human Oral Microbe Identification Microarray; NERD, non-erosive reflux disease; NGS, next-generation sequencing; RE, reflux esophagitis.

## Data Availability

Not applicable.
